# Anaerobic degradation of polycyclic aromatic hydrocarbons

**DOI:** 10.1128/aem.02268-24

**Published:** 2025-04-02

**Authors:** Isabelle Heker, Nadia A. Samak, Yachao Kong, Rainer U. Meckenstock

**Affiliations:** 1 Institute for Environmental Microbiology and Biotechnology, Aquatic Microbiology, Faculty of Chemistry, University of Duisburg-Essen 119884 , Essen, Germany; Kyoto University, Kyoto, Japan

**Keywords:** PAH degradation, anaerobic degradation, polycyclic aromatic hydrocarbons, sulfate-reducing bacteria, bioremediation, environmental microbiology

## Abstract

Polycyclic aromatic hydrocarbons (PAHs) are ubiquitous and toxic pollutants in the environment that are mostly introduced through anthropogenic activities. They are very stable with low bioavailability and, because aerobic degradation is mostly limited in aquifers and sediments, often persist in anoxic systems. In this review, we elucidate the recent advances in PAH degradation by anaerobic, mostly sulfate-reducing cultures. The best-studied compound is naphthalene, the smallest and simplest PAH, which often serves as a model compound for anaerobic PAH degradation. In recent years, three-ring PAHs have also shifted into focus, using phenanthrene as a representative compound. Anaerobic degradation of PAHs has to overcome several biochemical problems. First, non-substituted PAHs have to be activated by carboxylation, which is chemically challenging and proposed to involve a 1,3-cycloaddition with a UbiD-like carboxylase and a prenylated flavin cofactor. The second key reaction is to overcome the resonance energy of the ring system, which is performed by consecutive two-electron reduction steps involving novel type III aryl-CoA reductases belonging to the old-yellow enzyme family. In naphthalene degradation, a type I aryl-CoA reductase is also involved in reducing a benzene ring structure. The third key reaction is the ring cleavage, involving β-oxidation-like reactions in cleaving ring I of naphthalene. Ring II, however, is opened by a novel lyase reaction at a tertiary, hydroxylated carbon atom. These principles are explained using examples of anaerobic naphthalene and phenanthrene degradation to give an overview of recent advances, from the initial activation of the molecules to the complete degradation to CO_2_.

## INTRODUCTION

### Polycyclic aromatic hydrocarbons (PAHs) in the environment

Aromatic hydrocarbons are a class of organic pollutants occurring in nearly all environments worldwide and imposing a wide range of negative effects on habitats as well as humans and animals. Practically all aromatic hydrocarbons exhibit adverse health effects, ranging from carcinogenicity and acute toxicity in humans to general toxic and mutagenic effects on all life forms (1–5).

Many aromatic compounds occur naturally in the environment in minor amounts, originating from volcanic eruptions and natural oil seeps, or production as metabolites or signaling and defense molecules by some plants, fungi, or a few species of termites (6–9).

Anthropogenic point sources where aromatic hydrocarbons are discharged into the environment include landfills, previous industrial sites such as coal gasification factories, leaking pipelines and tanks, and spills during the transport of chemicals or oil. Diffuse sources are mostly associated with the incomplete combustion of wood, fossil fuels, and coal. Such contaminations are widespread and can move with the groundwater flow, and it is practically impossible to totally remove them (10, 11).

Aromatic hydrocarbons can be classified into monocyclic hydrocarbons (BTEX—benzene, toluene, ethylbenzene, and xylene) and PAHs. PAHs are more diverse, including various aromatic compounds with more than one aromatic ring, for example, naphthalene with two rings or phenanthrene and anthracene with three rings. Naphthalene is often used as a model compound as it is the smallest and simplest PAH. Very little is known about the degradation of PAHs with three or more rings, but the pathways are expected to be very similar to naphthalene degradation. For this reason, the review will focus on naphthalene degradation exemplarily, and the bigger PAHs will be discussed briefly at the end of this manuscript.

Most PAHs have a very low water solubility, of which naphthalene exhibits one of the highest, with approximately 0.25 mM at 25°C (12). The water solubility of PAHs decreases with increasing number of aromatic rings. Due to the stability of the molecules with 10 or more delocalized π-electrons, the low water solubility, and the high tendency to adsorb to sediments or soils, the bioavailability of PAHs is low (13–15).

Aerobic degradation of aromatics is well studied and will not be addressed in this review (16–18). The most relevant difference between aerobic and anaerobic degradation of aromatic hydrocarbons is the use of oxygenases and molecular oxygen in aerobic microorganisms to activate the molecule by hydroxylating and finally cleaving the aromatic ring. All these reactions are no option in anoxic environments (19). However, oxygen gets quickly depleted during aerobic degradation, and contaminated aquifers and sediments turn anoxic upon small contaminant loads, opening an ecological niche for anaerobic degraders (20).

### PAHs as carbon and electron source

First experiments showing anaerobic degradation of aromatic hydrocarbons were carried out with environmental samples and sewage sludge, but only a few enrichments or pure cultures were obtained (21–27). This could be due to the slow growth of the cultures with doubling times ranging from a few weeks to several months, as well as the small growth yields (20, 28).

Sulfate is a common electron acceptor used during anaerobic PAH degradation (22, 23, 29–33), but denitrification (21), methanogenesis (34, 35), and iron reduction (31, 36, 37) have also been shown. The first evidence of anaerobic degradation of naphthalene or higher PAHs under sulfate-reducing conditions was found in soil samples (32, 38). In the same year, carboxylic acids (2-naphthoic acid and 2-phenanthroic acid, respectively) were found as intermediates of naphthalene and phenanthrene degradation in an enrichment culture from contaminated harbor sediment, indicating carboxylation as the initial activation reaction of naphthalene and phenanthrene (22). The first sulfate-reducing, pure culture degrading naphthalene “NaphS2” belongs to the family *Deltaproteobacteraceae* of the former δ-proteobacteria, now phylum Desulfobacteriota (23, 39). Another sulfate-reducing enrichment culture “N47” growing with naphthalene or 2-methylnaphthalene contained a sulfate-reducing microorganism also belonging to the *Deltaproteobacteraceae* as the most abundant organism in the culture (40). The corresponding 16S rRNA gene of the naphthalene-degrading bacterium in culture N47 is distantly related to the sequence of *Desulfobacterium cetonicum* (GenBank accession number AJ237603) with 93% sequence similarity (41). Organisms closely related to N47 were found in naphthalene-degrading enrichment cultures from four different sites in Germany and the Czech Republic using groundwater and aquifer sediments as inoculum, indicating that such sulfate reducers seem to be important for anaerobic PAH degradation (42).

Some of the first experiments showing degradation of naphthalene also showed degradation of phenanthrene in environmental samples (22, 32). In the following decades, naphthalene degradation was investigated at the enzyme level, while phenanthrene degradation was targeted in only very few studies. Three enrichment cultures with phenanthrene were established with sulfate as an electron acceptor (43–45). Recently, a pure culture degrading phenanthrene was reported using nitrate as an electron acceptor (46), and a strain description and culture-bank deposition are eagerly anticipated by the scientific community.

TRIP_1 culture was enriched from contaminated soil near Pitch Lake, Trinidad, and could grow anaerobically with phenanthrene as the sole carbon and electron source (44). Metagenomic analysis of the TRIP_1 culture degrading phenanthrene with sulfate as an electron acceptor revealed that the dominant organism belongs to the genus *Desulfatiglans* and shares 94% similarity with *Desulfatiglans anilini* strain AK1 (47) and 87% similarity with the Phe4A strain from the phenanthrene-degrading culture Phe4 (43).

Rothermich et al. reported degradation of higher-molecular weight PAHs up to five rings in harbor sediments contaminated with coal tar. Degradation of larger PAHs proceeds even slower, but there was a significant reduction of naphthalene (two rings), phenanthrene and anthracene (three rings), pyrene, benzo[a]anthracene and chrysene (four rings), benzo[a]pyrene (five rings) as well as acenaphthene, fluorine, and fluoranthene (27). For example, phenanthrene, anthracene, pyrene, and benz[a]pyrene were degraded at rates of 0.55, 0.65, 0.23, and 0.33 µmol kg^−1^ day^−1^, respectively, where kg refers to the dry mass of sediment used in the incubations. Degradation of some of these substrates could be confirmed in sediments from contaminated rivers under sulfate-reducing conditions, as well as under methanogenic and nitrate-reducing conditions (26) and in enrichment cultures from gas-condensate contaminated sediments under methanogenic conditions (48–50). Hence, there seems to be potential for anaerobic microbial degradation of a wider range of PAHs in the environment.

**Fig 1:**
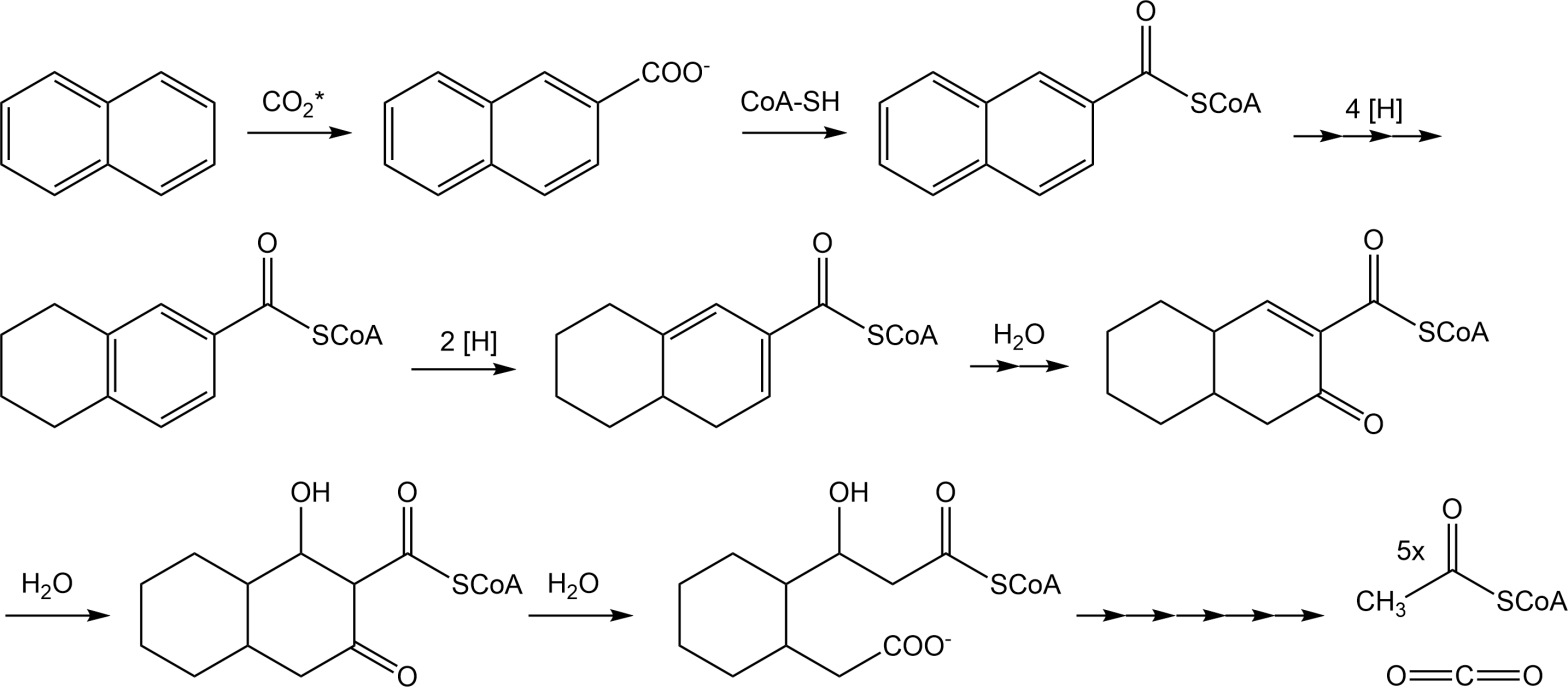
Schematic overview of naphthalene degradation as proposed for the sulfate-reducing culture N47. Naphthalene is activated by carboxylation and CoA-ligation. The carboxyl donor is either CO_2_ or bicarbonate and is marked with an asterisk in the figure. Then, the aromatic rings are reduced first on the non-substituted ring and then on the ring carrying the carboxyl group. After the reduction of three double bonds, consecutive hydration of the remaining double bonds, and oxidation of the hydroxyl to keto groups, the ring is cleaved and split into five molecules of acetyl-CoA and one CO_2_.

The anaerobic degradation of PAHs has to overcome several biochemical problems caused by the chemical stability of aromatic rings. First, the PAH has to be activated to a carboxylic acid to obtain a “biochemical handle” after which subsequent activation and reduction reactions can convert the molecule to a non-aromatic intermediate. This carboxylation reaction is chemically very demanding since a proton has to be replaced by a CO_2_ molecule at the poorly reactive aromatic ring. PAHs with alkyl side chains can be activated by fumarate addition to a methyl group or alkyl side chain, which has been demonstrated for degradation of 1- and 2-methylnaphthalene (24, 41, 51–53). Second, once activated, the resonance energy has to be overcome for cleaving the ring system. As oxidation of the ring is not an option, anaerobes reduce the ring before cleavage. Third, the ring cleavage itself occurs at tertiary carbon atoms, which requires non-canonical hydrolytic processes. In the following, we will go through the different biochemical steps and challenges to oxidize PAHs as carbon and electron sources in the absence of molecular oxygen. A general outline of a naphthalene degradation pathway as proposed for the culture N47 is shown in Fig. 1.

## ACTIVATION REACTIONS OF NON-SUBSTITUTED PAHs

### Carboxylation of naphthalene

The initial activating step in the degradation of non-substituted PAHs is a carboxylation to its corresponding carboxylic acid. This was first proven by the identification of ^13^C-labeled 2-naphthoic acid and phenanthroic acid from microcosms degrading naphthalene or phenanthrene that were amended with ^13^C-bicarbonate (22). The naphthalene-degrading enrichment culture N47 was found to accumulate 2-naphthoic acid, further supporting carboxylation as the activating step (40). It turned out that all naphthalene-degrading microorganisms found so far carboxylate the ring system in position 2 producing 2-naphthoic acid (20).

The carboxylation of naphthalene by culture N47 could be measured for the first time in enzyme assays using crude cell extracts and was found to be ATP- and biotin-independent, not dependent on divalent cations, and highly oxygen-sensitive (54). In contrast, Kölschbach et al. measured a fivefold increase in naphthalene carboxylation when ATP was added to assays with cell-free extracts of the same culture (55), and the authors of this review found a strong ATP-dependency of the same carboxylase reaction in recent studies (56). Hence, the carboxylation of naphthalene seems to depend on ATP, although it is not clear yet if this is due to a direct ATP utilization of the carboxylase or indirectly via coupling to the next enzyme reaction in the pathway, the 2-naphthoate:CoA ligase (see below). The free energy of the carboxylation reaction of naphthalene to naphthoic acid under standard conditions was calculated with the group contribution model and was found to be slightly endergonic with +14 kJ mol^−1^ (20, 54).

The results of the enzyme assays correspond well with proteogenomic studies which showed a twofold upregulation of a possible subunit of a naphthalene carboxylase in the genome of N47 during growth with naphthalene compared to 2-methylnaphthalene (57). This carboxylase showed 48% similarity to the anaerobic benzene carboxylase from the iron-reducing enrichment “strain BF” (58, 59) and 45% similarity to the α-subunit of phenylphosphate carboxylase from *Aromatoleum aromaticum* EbN1 (56, 60, 61) and belongs to the family of UbiD-like (de)carboxylases (56, 57, 62, 63). Other subunits of the naphthalene carboxylase also showed sequence identities of up to 48% to AbcA, the α-subunit of anaerobic benzene carboxylase of the benzene-degrading strain BF, and up to 46% identity to PpcA, the α-subunit of the phenylphosphate carboxylase of *Thauera aromatica* (57, 59, 64). In the naphthalene-degrading culture NaphS2, DiDonato et al. identified two genes with sequence similarities to 3-octaprenyl-4-hydroxybenzoate carboxylase that were upregulated during growth with naphthalene and possibly code for naphthalene carboxylases (65).

Naphthalene carboxylase of N47 is proposed to form a high-molecular-mass enzyme complex of approx. 750 kDa as shown by pull-down experiments with blue native gels and N47 cell extracts. It is encoded in one gene cluster with a size of 8.9 kb containing nine genes (N47_K27540 – N47_K27460) (57). Four of the genes encode subunits with a similarity to the 3-octaprenyl-4-hydroxybenzoate decarboxylase (UbiD) from *Escherichia coli* (N47_K27540, N4_K27500, N47_K27480, and N47_K27460) (57), placing naphthalene carboxylase in the rapidly expanding group of UbiD-like (de-)carboxylases (62, 63, 66–69). In strain NaphS2, a gene cluster containing the genes NPH_5855 to NPH_5865 was proposed to encode a naphthalene carboxylase based on enzyme similarities. Some of these genes were upregulated during the growth of NaphS2 on naphthalene compared to benzoate and also show sequence similarities to UbiD and the N47 naphthalene carboxylase (65).

Members of the UbiD enzyme family use a prenylated flavin mononucleotide cofactor (prFMN, compound **A** in Fig. 1) that was first detected in the homologous fungal enzyme ferulic acid decarboxylase (Fdc1) from *Aspergillus niger* (68, 70). prFMN is supplied to the decarboxylase by a flavin prenyltransferase (UbiX in *E. coli* and Pad1 in *A. niger*) (68). prFMN can be prepared in several different forms, including a radical form, an iminium, and a ketimine, while the iminium form is suggested to be the catalytically active species (66, 68, 71). The novel cofactor was also detected in other UbiD-like (de-)carboxylases such as phthaloyl-CoA decarboxylases from *Thauera chlorobenzoica* or *Desulfosarcina cetonica* (72, 73), among others.

**Fig 2:**
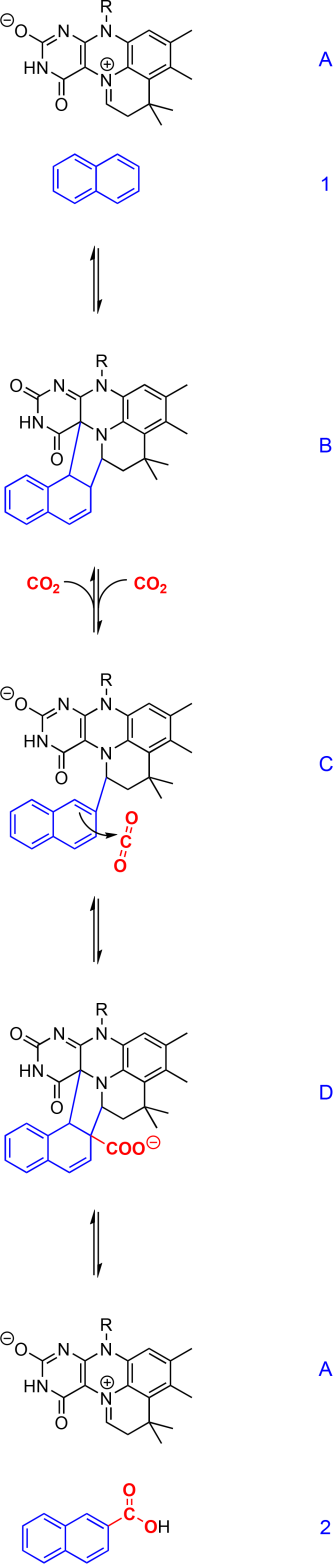
Proposed mechanism of naphthalene carboxylation catalyzed by the naphthalene carboxylase of strain N47 using prFMN as co-factor (56). Naphthalene is depicted in blue and CO_2_ in red and bold print. RCOO^−^ represents a weak base that facilitates proton abstraction by an amino acid residue in the active site, such as glutamic acid in Fdc1 from *Aspergillus niger* (68).

The mechanism of naphthalene carboxylation was proposed recently based on stable isotope labeling in enzyme assays which suggest a two-step reaction with a stable intermediate and the reversibility of at least the latter reaction step (56). Quantum mechanical gas phase calculations were performed to elucidate which of the possible mechanisms appears most likely (Fig. 2). The reaction starts with a 1,3-dipolar cycloaddition of the naphthalene C1 and C2 carbon atoms to the cofactor prFMN. Ring one of naphthalene is dearomatized in the resulting intermediate (compound **B** in Fig. 2). In the next step, deprotonation of naphthalene C2 leads to re-aromatization of the ring, and the bond between naphthalene C1 and prFMN C4a is broken. The proton abstraction is likely facilitated by amino acid residues in the active site acting as weak bases (68). The resulting intermediate (compound **C** in Fig. 2) is relatively stable and can also be formed by a backward reaction from 2-naphthoate, which was proven by an isotope exchange of the carboxyl group of ^12^C-naphthoate and ^13^C-bicarbonate in assays with cell-free extract of culture N47 and no ATP. This also indicates the reversibility of the later steps of the carboxylation reaction as well as their ATP-independency.

Compound **C** undergoes an electrophilic addition of CO_2_ at position C2 that also re-establishes the ring system between naphthalene C1 and C2 and prFMN (compound **D** in Fig. 2). After that, the bonds between the formed 2-naphthoate and the prFMN are cleaved, and the aromatic system is re-established, setting the cofactor free for the next reaction (56).

### Activation to CoA-thioester

**Fig 3:**
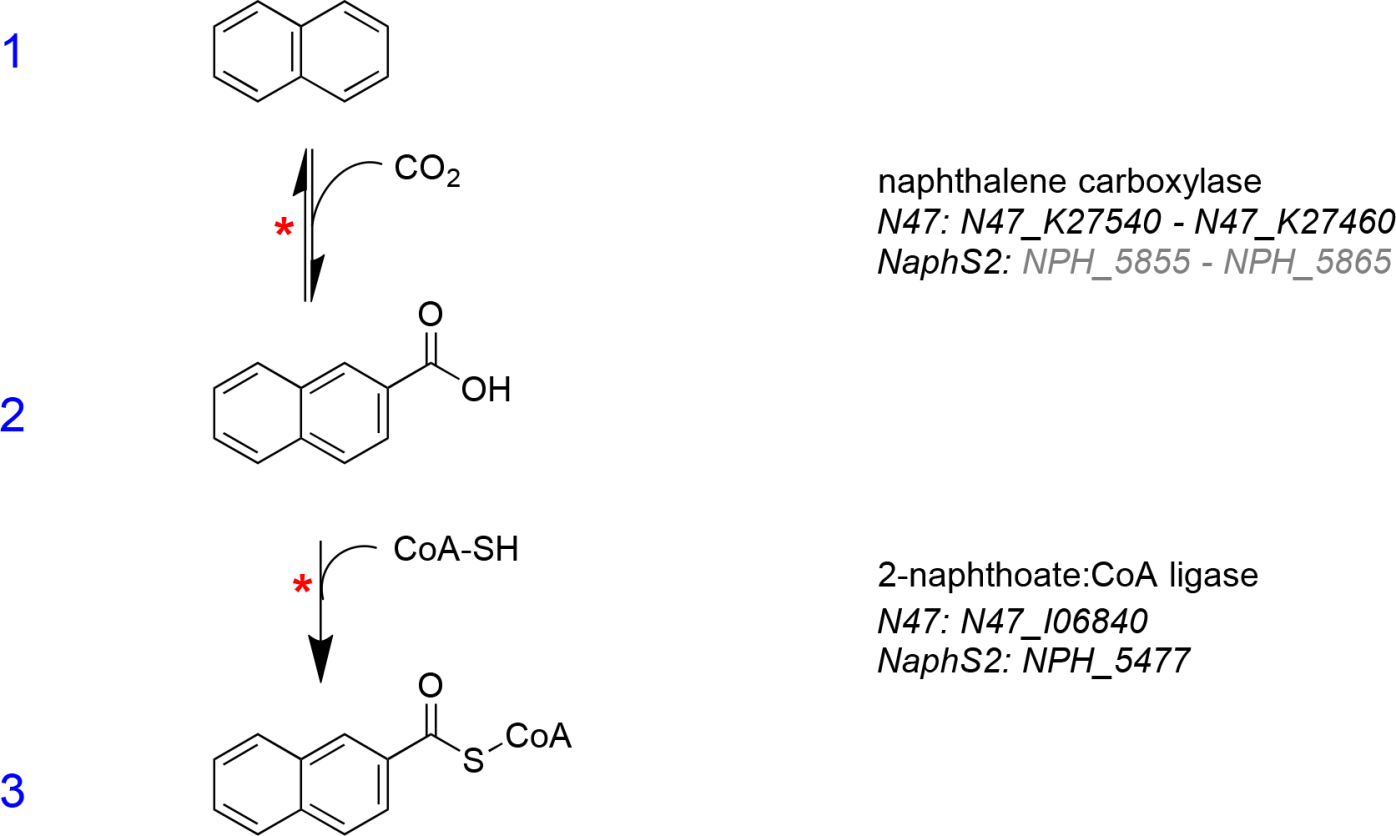
Naphthalene aromatic ring activation via carboxylation in position 2 by the naphthalene carboxylase and subsequent CoA-thioesterification catalyzed by the 2-naphthoate:CoA ligase. A red asterisk (*) marks reactions that were proven in enzyme assays using cell-free extracts. Putative genes marked in gray are only based on enzyme similarities and therefore not very certain.

After carboxylation, the PAH carboxylic acids undergo a thioesterification reaction with coenzyme A (CoA). This reaction could be measured in crude cell extracts of strain N47 grown with naphthalene (2-naphthoate:CoA ligase) and in crude cell extracts of strain TRIP_1 grown with phenanthrene (2-phenanthroate:CoA ligase) (39, 44, 61, 74). An overview of the naphthalene carboxylation with the following CoA-thioesterification catalyzed by the 2-naphthoate:CoA ligase is shown in Fig. 3. Recently, both reactions could also be measured in cell-free extracts, and the enzymes could be characterized by our group (74). The 2-naphthoate:CoA ligase depends on magnesium ions and ATP and is not sensitive to oxygen (75). Both N47 and NaphS2 genomes contain several candidate ligase genes, the most likely ones were proposed to be N47_I06840 in N47 (55, 75) and NPH_5477 in NaphS2 (55, 65, 75).

Thioesterification can serve several purposes in the metabolism. For example, in the anaerobic benzoate-degrading organism *Rhodopseudomonas palustris*, this reaction helps to accumulate the substrate benzoate in the cell by maintaining a downhill gradient in the cell cytoplasm compared to the substrate concentration outside the cell (76). Furthermore, the polar structure and size of the CoA thioester prevent substrate loss by diffusion through the membrane (77). The CoA thioester also facilitates further reduction steps due to its electron-withdrawing property toward the CoA group and ability to stabilize likely ketyl intermediates (78).

All aryl-carboxylate:CoA ligases appearing in anaerobic PAH degradation pathways known to date belong to class I of the adenylate-forming enzyme superfamily. This class is composed of two major types, benzoate:CoA ligase-like enzymes and phenylacetate:CoA ligase-like enzymes. Both the 2-naphthoate:CoA ligase and the 2-phenanthroate:CoA ligase belong to the phenylacetate:CoA ligase-like type. For further details on this widespread family of enzymes, we refer the reader to a recent review article (79).

The mechanism of aryl-CoA ligases consists of two steps: in the first step, an aryl-adenylate intermediate is formed by nucleophilic attack of the negatively charged oxygen of the carboxyl group on the α-phosphorus of ATP, releasing pyrophosphate. In the second step, the thiol group of CoA performs a nucleophilic attack on the carboxyl carbon of the adenylate intermediate with AMP as the leaving group, forming a thioester (79–82).

Substrate specificity varies widely among aryl-carboxylate:CoA ligases. The 2-naphthoate:CoA ligase substrate spectrum is rather broad; it can convert fluorinated naphthoates at a similar rate as 2-naphthoate and hydroxylated naphthoates at a lower rate (M. Arnold and R. U. Meckenstock, unpublished results).

## RING REDUCTION STEPS DURING NAPHTHALENE DEGRADATION

Microbiological and biochemical investigations with the model PAH compound naphthalene indicated that the anaerobic degradation of PAHs includes several biochemically unprecedented reactions, such as the ATP-independent and ATP-dependent reductive de-aromatization of 2-naphthoyl-CoA intermediates (83). Since anaerobes cannot rely on oxygen as a reactive co-substrate, the resonance energy of the aromatic ring of PAHs is overcome by reduction with dearomatizing aryl-CoA reductases (84, 85).

Three distinct aromatic ring reduction strategies are known so far. The first strategy is the aromatic ring reduction by type I aryl-CoA reductases. This type of reductase is sensitive to oxygen and ATP-dependent (86). They catalyze the reduction of benzoyl-CoA in the presence of magnesium and ATP to produce cyclohexa-1,5-diene-1-carboxyl-CoA with ferredoxin as a natural electron donor. Type I aryl-CoA reductases are characterized by an oligomeric structure of four subunits a, b, c, and d with a molecular mass of 48, 45, 38, and 32 kDa, respectively. They contain iron-sulfur clusters and FAD (87). This type of enzyme can be found in facultative anaerobic bacteria such as *R. palustris*, *T. aromatica*, or *Azoarcus* strain CIB (88–90).

The second strategy is the aromatic ring reduction by the oxygen-sensitive type II aryl-CoA reductases, which differ from type I aryl-CoA reductases in their ATP-independency (91). They are responsible for the ring reduction by an electron bifurcation mechanism in the majority of strict anaerobes. The reduction reaction by type I aryl-CoA reductases becomes exergonic by the transfer of one low-potential electron from ferredoxin to the substrate and the transfer of another electron to NAD(P)^+^ as a high-potential electron acceptor (92). The type II aryl-CoA reductase of *Geobacter metallireducens* is encoded by bamBCDEFGHI gene clusters (93). The encoded enzyme is a big enzyme complex composed of eight subunits. The individual proteins of the bamBCDEFGHI complex are similar to soluble components of NADH:quinone oxidoreductases (BamGHI), electron transfer components of hydrogenases (BamC and BamF), soluble heterodisulfide reductases (BamDE), and to molybdenum- or tungsten-containing aldehyde:ferredoxin oxidoreductases (BamB) (94). Type II aryl-CoA reductases were found in the iron reducer *Geobacter metallireducens* and the sulfate reducer *Desulfococcus multivorans* (94–96).

The third strategy is the aromatic ring reduction by the newly discovered type III aryl-CoA reductase, which has only been found for anaerobic PAH degradation (97). Type III aryl-CoA reductases belong to the old-yellow enzyme family (OYE), which are composed of three domains binding flavin adenine dinucleotide (FAD), flavin mononucleotide (FMN), and a [4Fe-4S] cluster (98). Enzymes belonging to the OYE family are ATP-independent and oxygen-insensitive. They reduce 2-naphthoyl-CoA without energetic coupling to an exergonic reaction.

**Fig 4:**
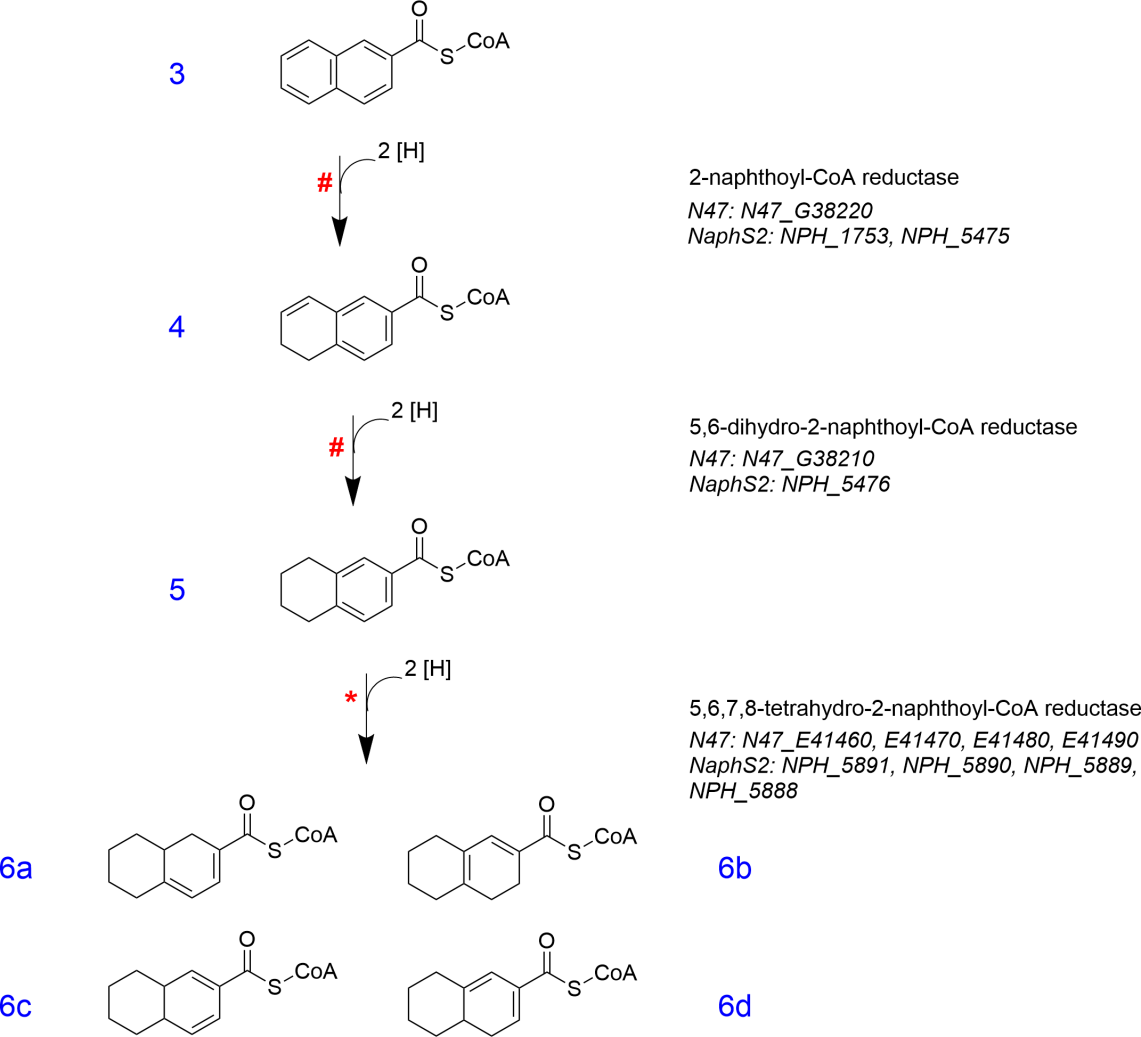
Stepwise reduction of the aromatic rings of naphthalene in the enrichment culture N47. 2-naphthoyl-CoA (**3**) is reduced by the ATP-independent 2-naphthoyl-CoA reductase (NCoA-R) to produce 5,6-dihydro-2-naphthoyl-CoA (**4**). Compound **4** is reduced to 5,6,7,8-tetrahydro-2-naphthoyl-CoA (**5**) by the ATP-independent 5,6-dihydro-2-naphthoyl-CoA reductase (DNCoA-R), which is then reduced to the final reduction product, hexahydro-2-naphthoyl-CoA (**6**) by the ATP-dependent 5,6,7,8-tetrahydro-2-naphthoyl-CoA reductase (THNCoA-R). A red hash symbol (#) indicates that this reaction step was proven in enzyme assays with heterologously expressed enzymes, and a red asterisk (*) marks that this reaction was shown in enzyme assays with cell-free extracts of culture N47.

The anaerobic degradation steps of naphthalene have been studied by elucidating the downstream degradation intermediates of the sulfate-reducing naphthalene-degrading enrichment culture N47 and the pure culture NaphS2 (23, 40). In culture N47, the anaerobic degradation of 2-naphthoyl-CoA proceeds via a multistep process using two successive reduction steps with newly discovered type III aryl-CoA reductases at the non-substituted ring, followed by one reduction step at the substituted ring employing an ATP-dependent type I aryl-CoA reductase (28, 83). The first reductase, the ATP-independent 2-naphthoyl-CoA reductase (NCoA-R), has been studied in the sulfate-reducing culture N47 and the pure culture NaphS2. There were three potential genes encoding NCoA-R found in cultures N47 (N47_G38220) and NaphS2 (NPH_1753 and NPH_5475) (97), which were heterologously expressed in *E. coli* with a C-terminal double Strep-tag and characterized (98). The three enzymes were found to be responsible for the catalytic conversion of 2-naphthoyl-CoA to 5,6-dihydro-2-naphthoyl-CoA (compound **4** in Fig. 4). The preferable artificial electron donor for the reduction assays was dithionite, which has a very negative standard redox potential of *E*^ο′^ = −493 mV, with specific activities for 2-naphthoyl-CoA reduction in the range of 22–30 nmol min^−1^ mg^−1^ for N47_G38220, NPH_1753, and NPH_5475. The gene products of the N47_G38220, NPH_1753, and NPH_5475 subunits have a predicted size of 76.5, 77, and 79.3 kDa, respectively. The three NCoA-R enzymes contain stoichiometric amounts of FMN (0.86, 0.94, and 0.79 per subunit of N47_G38220, NPH_1753, and NPH_5475, respectively), FAD (0.88, 0.9, and 0.83), and one [4Fe-4S] cluster (98, 99). NCoA-R expressed from N47_G38220 was intensively characterized, and it was found that NCoA-R uses a low-potential one-electron donor for the two-electron dearomatization of the 2-naphthoyl-CoA below the redox limit of *E*^ο′^ = −493 mV (99). The electrochemical perspective of the reduction of 2-naphthoyl-CoA using NCoA-R revealed that one-electron reduction of both iron-sulfur cluster and FAD occurs first, then two single electrons are transferred to the FMN. After protonation, these electrons form a hydride, which is then transferred to 2-naphthoyl-CoA to overcome the resonance energy of the aromatic system (99). NCoA-R catalyzes the reduction reaction of 2-naphthoyl-CoA through a hydride addition in a CoA-thioester-dependent Meisenheimer complex-analogous manner (100).

The next step in the reaction sequence is the reduction of 5,6-dihydro-2-naphthoyl-CoA to 5,6,7,8-tetrahydro-2-naphthoyl-CoA (THNCoA, compound **5** in Fig. 4) using the ATP-independent 5,6-dihydro-2-naphthoyl-CoA reductase (DNCoA-R) at a redox potential *E*^ο′^ = −375 using NADH as an electron donor (98). The potential genes encoding DNCoA-R were found in culture N47 (N47_G38210) and NaphS2 (NPH_5476) with 33–34% amino acid sequence identity to NCoA-R. The two enzymes were heterologously expressed in *E. coli* with a C-terminal double Strep-tag and characterized (98). The preferable electron donor for the enzyme assays was dithionite with specific activities of 625 and 95 nmol min^−1^ mg^−1^ for N47_G38210 and NPH_5476, respectively. Reduction with NCoA-R or DNCoA-R also worked with 75% reduced methyl viologen as an electron donor reducing 5,6-dihydro-2-naphthoyl-CoA to 5,6,7,8-tetrahydro-2-naphthoyl-CoA. The theoretical molecular weights of the enzymes N47_G38210 and NPH_5476 are 78 and 76.7 kDa, respectively. The native molecular weights were not determined (98). FMN, FAD, and iron content of DNCoA-R are not known due to the very low amount of protein expressed by *E. coli,* but the amino acid alignment with OYE enzymes revealed the presence of the binding motifs of FMN, FAD, and an iron-sulfur cluster. Reduction of 5,6-dihydro-2-naphthoyl-CoA to 5,6,7,8-tetrahydro-2-naphthoyl-CoA proceeds via a protonation-initiated mechanism (100). The mechanism involves isomerization by transient deprotonation of carbon number 5 of the 5,6-dihydro-2-naphthoyl-CoA to facilitate direct hydride transfer to carbon number 7 with resonance stabilization by the CoA ester (100).

After two consecutive reduction steps of the non-substituted ring I of naphthoic acid, the remaining double bond in ring I is further reduced by the ATP-dependent 5,6,7,8-tetrahydro-2-naphthoyl-CoA reductase (TNCoA-R), producing hexahydro-2-naphthoyl-CoA (HHNCoA, compound **6** in Fig. 4) with unknown position of the diene moiety (97). Potential genes encoding TNCoA-R were found in culture N47 (N47_E41460, E41470, E41480, and E41490) and NaphS2 (NPH_5891, NPH_5890, NPH_5889, and NPH_5888). TNCoA-R belongs to class I of the aryl-CoA reductase family (similar to benzoyl-CoA reductase) and is oxygen-sensitive with a half-life time of 30 s to 1 min when exposed to air. The reduction reaction was maximal with 1 mM NADH as an electron donor and showed a specific activity of 0.7 nmol min^−1^ mg^−1^ (83, 97). The reduction also occurred with other electron donors such as 1 mM NADPH, 2 mM sodium dithionite, and 2 mM titanium(III)citrate but only with reduction rates of 69%, 41%, and 29%, respectively (97). It is surprising that NADH can be used as an electron donor because the ATP-dependent benzoyl-CoA reductase prefers reduced ferredoxin or NADPH combined with NADPH:ferredoxin-oxidoreductase (86). While 2-naphthoyl-CoA has a higher conjugation of the aromatic ring system and can be reduced without ATP, breaking the aromaticity of the remaining benzene ring obviously needs ATP, similar to benzoyl-CoA reduction. The extremely slow growth rate of N47 with naphthalene as electron source could be due to the ATP hydrolysis that occurs during THNCoA reduction. Remarkably, in the genomes/metagenomes of cultures N47 and NaphS2, it can be observed that the genes responsible for potential NAD(P)H-dependent OYE oxidoreductases and ferredoxins are positioned in close proximity to genes resembling class I benzoyl-CoA reductases such as the TNCoA-R. This arrangement suggests their potential involvement in NAD(P)H-dependent electron transfer to THNCoA via ferredoxin following the same biochemical principle as in benzoyl-CoA reduction (83, 97). It is noteworthy that the enzyme assays of THNCoA-R were performed with cell-free extracts of the culture N47, and the enzyme was not heterologously expressed in *E. coli*. Hence, cell-free extracts of N47 probably contained the ferredoxin that is required as a natural electron donor (83).

In earlier studies with culture N47, the detection of the metabolites octahydro-2-naphthoic acid and decahydro-2-naphthoic in culture supernatants suggested that the stepwise reduction would proceed after the formation of hexahydro-2-naphthoyl-CoA (compound **6** in Fig. 4) (97). The biochemical studies, however, indicated that these compounds were secreted as dead-end products and not as natural intermediates (83, 97). This was supported by enzyme assays where NAD^+^ was added to the enzymatic conversion of THNCoA to HHNCoA by N47 cell-free extracts, and β-hydroxyoctahydro-2-naphthoyl-CoA (compound **7** in Fig. 6) was produced, which indicates a hydration of HHNCoA but not octahydro-2-naphthoyl-CoA. Hence, the downstream degradation pathway of naphthalene does not proceed via benzoyl-CoA derivatives but via metabolites with a cyclohexane skeleton (83).

The ring reduction pathway discovered in culture N47 seems to be similar in other anaerobic naphthalene degraders such as NaphS2. A gene cluster (*NPH_5472–76*) encoding for five oxidoreductase genes was found in the genome of strain NaphS2. Two genes, NPH_5475 and NPH_5476, were already mentioned above, and their function toward 2-naphthoyl-CoA aromatic ring reduction is similar to NCoA-R and DNCoA-R, respectively. The function of the remaining three genes from the gene cluster is still unknown and needs further investigation. Another gene cluster (*ncrABCD*), which has homology to *Azoarcus bzdNOPQ (NPH_5888*, UniProt) and expresses 2-naphthoyl-CoA reductase (NPH_5891, UniProt), was also discovered in NaphS2 pure culture when grown on naphthalene (65).

## RING CLEAVAGE PATHWAYS

### Ring cleavage pathway of naphthalene

After naphthalene has undergone carboxylation, CoA ligation, and three consecutive two-electron reduction reactions, hexahydro-2-naphthoyl-CoA (HHNCoA) is generated with unknown position of the diene double bonds (101). The latter must now undergo two ring cleavage steps leading to acetyl-CoA and pimeloyl-CoA.

**Fig 5:**
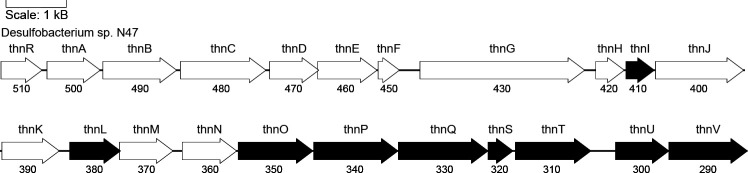
The *thn*-operon in the genome of the enrichment culture N47. Genes encoding enzymes with confirmed functions are shown in black, and those with unknown functions are shown in white. The full gene locus tag follows the format “N47_E41XXX”; in this figure, gene locus tags are abbreviated to the last three digits (e.g., “510” for “N47_E41510”).

The genes encoding 5,6,7,8-tetrahydro-2-naphthoyl-CoA reductase are surrounded by a cluster of genes coding for β-oxidation-like enzymes (Fig. 5), which are expressed in 2-methylnaphthalene-grown N47 cells and naphthalene-grown NaphS2 cells (41, 61, 65). All 22 genes within this cluster are co-transcribed and form an operon named the *thn*-operon, in reference to the first substrate of the degradation steps catalyzed by this operon, 5,6,7,8-tetrahydro-2-naphthoyl-CoA (thn) (75). The *thn*-operon was recently demonstrated to be involved in downstream degradation steps (102).

### Cleavage of the first ring of naphthalene

**Fig 6:**
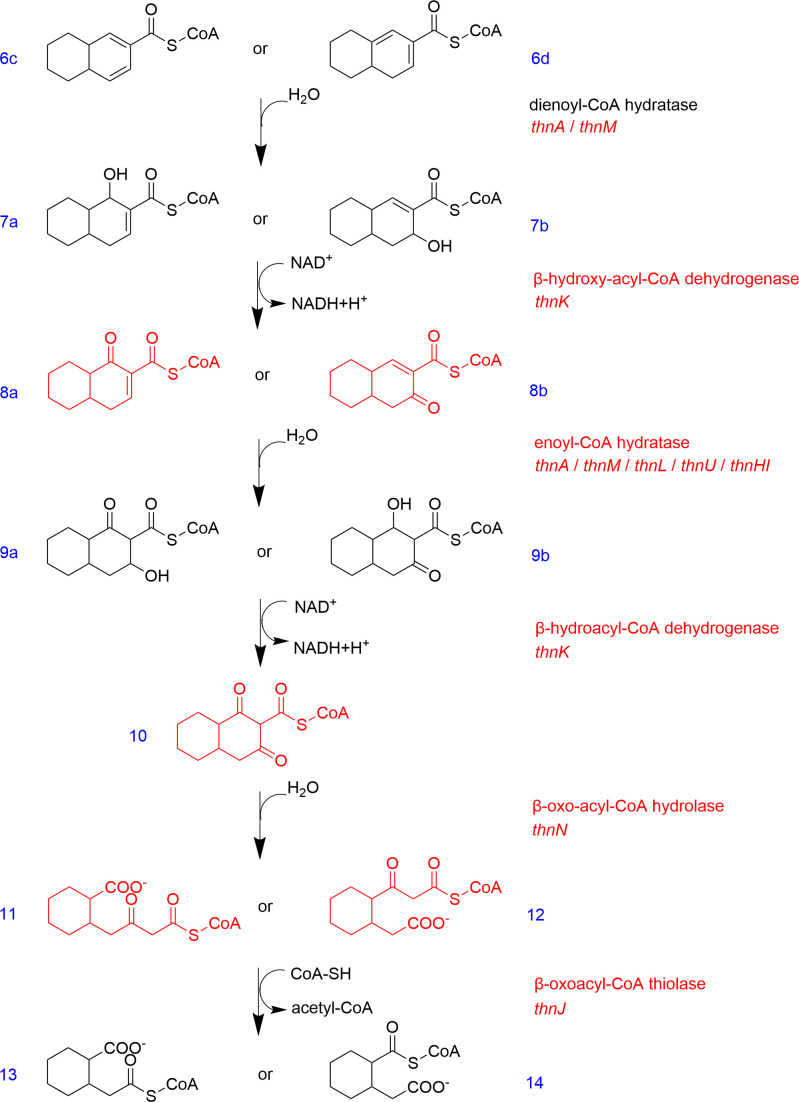
Proposed first ring-cleavage pathway of hexahydro-2-naphthoyl-CoA with double bonds in *α, β*- or *β′, γ′*-position (**6c** or **d**). Metabolites, enzymes, and genes in black have been proven in enzyme assays, and the ones in red are postulated. All reactions are measured with N47 cell-free extracts. The *β*-oxidation-like pathway is shown as two possible options since the conformation of hexahydro-2-naphthoyl-CoA is unknown so far. The pathway involves *β*-hydroxyoctahydro-2-naphthoyl-CoA (**7a** and **b**), *β*-oxooctahydro-2-naphthoyl-CoA (**8a** and **b**), *β*′-hydroxy-*β*-oxodecahydro-2-naphthoyl-CoA (**9a** and **b**) and 1,3-dioxodecahydro-2-naphthoyl-CoA (**10**). Cleavage of the first naphthalene ring results in the formation of 4-(2-carboxycyclohexyl)−3-oxobutyryl-CoA (**11**) or 3-(2-[carboxymethyl]cyclohexyl)−3-oxopropionyl-CoA (**12**), which is subsequently converted to 2-carboxycyclohexylacetyl-CoA (**13**) or 2-(carboxymethyl)cyclohexane-1-carboxyl-CoA (**14**). The pathway via intermediates **12** and **14** is proposed to be the correct one based on experimental results of the conversion of 2-(carboxymethyl)cyclohexane-1-carboxyl-CoA (**14**) by heterologously expressed enzyme.

The detection of the metabolite β-hydroxyoctahydro-2-naphthoyl-CoA (compound **7a** or **b** in Fig. 6) resulting from β-oxidation-like reactions of HHNCoA in the cell-free extract of N47 indicates a downstream pathway that involves water addition to HHNCoA (83). The hydratase reaction produces one of the two hypothetical products that could not be distinguished so far as a consequence of the different possible conformations of hexahydro-2-naphthoyl-CoA. The 1,4-addition of water to HHNCoA is in analogy to the benzoyl-CoA pathway in the denitrifying strain *T. aromatica* K172, where cyclohexa-1,5-diene-1-carboxyl-CoA is converted to 6-hydroxy-1-cyclohexene-1-carbonyl-CoA by dienoyl-CoA hydratase (Dch) (103, 104). In *R. palustris*, 1-cyclohexene-1-carbonyl-CoA (enoyl-CoA) is converted by the respective enoyl-CoA hydratase (BadK) to the 2-hydroxy intermediate (104). Within the *thn*-operon of the sulfate-reducing bacterium N47, seven potential hydratases/hydrolases are encoded, which are annotated as either crotonase-like hydratases (ThnA, ThnL, ThnM, and ThnU), MaoC-like dehydratases (ThnH and ThnI), or metallo-dependent hydrolases (ThnN) (Table 1). According to the phylogenetic analysis, all candidate hydratases are similar to enoyl-CoA hydratases but not to dienoyl-CoA hydratases, which is contradictory to the observation that the HHNCoA-hydratase in N47 acts on the dienoyl substrate.

**TABLE 1:** Genes encoded in the *thn*-operon in N47 and NaphS2 and their hypothetical functions^*a*^

Gene	ORF in N47		ORF in NaphS2	Hypothetical function
	Current naming	Previous naming (41)		
*thnR*	N47_E41510	ORF27	NPH_5886	Transcriptional regulator
*thnA*	N47_E41500	ORF28	NPH_5887	Enoyl-CoA hydratase/isomerase
*thnB*	N47_E41490	*ncrC*	NPH_5888	5,6,7,8-tetrahydro-2-naphthoyl-CoA reductase
*thnC*	N47_E41480	*ncrB*	NPH_5889	5,6,7,8-tetrahydro-2-naphthoyl-CoA reductase
*thnD*	N47_E41470	*ncrA*	NPH_5890	5,6,7,8-tetrahydro-2-naphthoyl-CoA reductase
*thnE*	N47_E41460	*ncrD*	NPH_5891	5,6,7,8-tetrahydro-2-naphthoyl-CoA reductase
*thnF*	N47_E41450	ORF33	NPH_5892	Ferredoxin
*thnG*	N47_E41430	ORF34	n.p.	Oxidoreductase
*thnH*	N47_E41420	ORF35	NPH_5893	MaoC family dehydratase
*thnI*	N47_E41410	ORF36	NPH_5894	MaoC family dehydratase
*thnJ*	N47_E41400	ORF37	NPH_5895	β-oxoacyl-CoA thiolase
*thnK*	N47_E41390	ORF38	NPH_5896	β-hydroxyacyl-CoA dehydrogenase
*thnL*	N47_E41380	ORF39	NPH_5897	Enoyl-CoA hydratase/hydrolase/isomerase
*thnM*	N47_E41370	ORF40	NPH_5898	Enoyl-CoA hydratase/isomerase
*thnN*	N47_E41360	ORF41	NPH_5899	Metallo-dependent hydrolase
*thnO*	N47_E41350	ORF42	NPH_5900	Acyl-CoA dehydrogenase
*thnP*	N47_E41340	ORF43	NPH_5902	CoA-transferase
*thnQ*	N47_E41330	ORF44	NPH_5903	AtuA-like lyase
*thnS*	N47_E41320	ORF45	NPH_5904	AtuA-like lyase
*thnT*	N47_E41310	ORF46	NPH_5907	Acyl-CoA dehydrogenase
*thnU*	N47_E41300	ORF47	NPH_5908	Enoyl-CoA hydratase/isomerase
*thnV*	N47_E41290	ORF48	NPH_5909	β-oxoacyl-CoA thiolase
*thnW*	n.p.		NPH_5901	β-oxoacyl-ACP reductase
*thnX*	n.p.		NPH_5905	β-oxoacyl-ACP reductase
*thnY*	n.p.		NPH_5906	β-hydroxyacyl-CoA dehydrogenase

^*a*^ n.p., not present in the gene cluster.

Weyrauch et al. investigated the substrate specificity of the seven hydratase/hydrolase candidates encoded within the *thn* operon, excluding ThnN, using various linear and cyclic substrate analogs. Their findings demonstrate that ThnU acts as a bifunctional enoyl-CoA hydratase and *β*-oxoacyl-CoA hydrolase with specificity for linear substrates, while ThnL functions solely as a hydrolase on cyclic substrates. Heteromeric ThnHI was characterized as an enoyl-CoA hydratase specific to linear compounds. Neither ThnA nor ThnM showed any activity toward the tested substrates (75). Recent studies have found that none of the six hydratases tested could hydrate hexahydro-2-naphthoyl-CoA (Y. Kong and R. U. Meckenstock, unpublished results). Given that ThnA and ThnM also showed no activity against the analogs, it is possible that heterologously expressed ThnA and ThnM were not properly folded. Consequently, the potential ability of ThnA or ThnM to catalyze the hydration of hexahydro-2-naphthoyl-CoA may have been overlooked due to incorrect protein conformation.

The proposed pathway would now follow the dehydrogenation of *β*-hydroxyoctahydro-2-naphthoyl-CoA producing *β*-oxooctahydro-2-naphthoyl-CoA (compound **8a** or **b** in Fig. 6). This reaction is most likely catalyzed by ThnK, the sole enzyme within the *thn*-operon annotated as a *β*-hydroxyacyl-CoA dehydrogenase. *β*-oxooctahydro-2-naphthoyl-CoA is then hydrated by an unidentified hydratase to *β*’-hydroxy-*β*-oxo-decahydro-2-naphthoyl-CoA (compound **9a** or **b** in Fig. 6) which can be detected as a metabolite in assays with cell-free extracts. The subsequent reaction should be a dehydrogenation reaction probably catalyzed by ThnK, followed by a ring hydrolysis reaction, producing the so far undetected intermediates 4-(2-carboxycyclohexyl)−3-oxobutyryl-CoA or 3-(2-[carboxymethyl]cyclohexyl)−3-oxopropionyl-CoA (compound **11** or **12** in Fig. 6). This inference is based on the detection of the downstream metabolite CoA-ester of cis-2-(carboxymethyl)cyclohexane-1-carboxylic acid (compound **13** or **14** in Fig. 6) and the proposed downstream pathway (101). These findings indicate that N47 applies a strategy for opening the naphthalene skeleton similar to benzoyl-CoA degradation in *T. aromatica* K172. This process begins with a diene compound that is hydrated by a dienoyl-CoA hydratase and then oxidized by an NAD^+^-dependent hydroxyacyl-CoA dehydrogenase, converting the 6-hydroxy group to a 6-oxo group. The remaining double bond is then hydrated, followed by ring cleavage through a combined hydratase-hydrolase reaction. However, the homologous gene of *oah* encoding 6-oxocyclohex-1-ene-1-carboxyl-CoA hydratase/hydrolase from *T. aromatica* cannot be found in the genomes of N47 and NaphS2. Conversion of *β*-oxooctahydro-2-naphthoyl-CoA (compound **8a** or **b** in Fig. 6) might therefore be performed by two separate enzymes, an enoyl-CoA hydratase and the subsequently acting ring-cleaving hydrolase.

The gene encoding the hydrolase responsible for cleaving the first ring of naphthalene has yet to be identified. Among the seven hydratase/hydrolase candidates (ThnA, ThnM, ThnL, ThnU, ThnH, ThnI, and ThnN) within the *thn*-operon, ThnL is the only enzyme exhibiting ring-cleaving hydrolase activity on the cyclic substrate analog 2-oxocyclohexanecarboxyl-CoA. It has been consequently postulated as the enzyme responsible for the ring-cleaving step during the anaerobic degradation of 2-naphthoyl-CoA (28). However, ThnL did not exhibit hydrolysis activity for catalyzing 1,3-dioxodecahydro-2-naphthoyl-CoA (compound **10** in Fig. 6) (Y. Kong and R. U. Meckenstock, unpublished results). As ThnN is the only candidate hydrolase that has not been tested for conversion of either analogs or the real intermediate (1,3-dioxodecahydro-2-naphthoyl-CoA), ThnN currently remains the best candidate for catalyzing the opening of the first ring of naphthalene. ThnN belongs to the amidohydrolase superfamily (105), which is characterized by a common TIM barrel fold and an active-site architecture enabling the deprotonation of water via a metal ion for subsequent nucleophilic attack on the substrate (106). A homologous protein, BamU, encoded by a gene cluster involved in anaerobic benzoate degradation in *Geobacter metallireducens*, shares 43% amino acid sequence similarity with ThnN, but its function remains unknown (95). Consequently, it still remains unclear whether ThnN is the responsible enzyme for hydrolyzing and opening the first ring of naphthalene.

**Fig 7:**
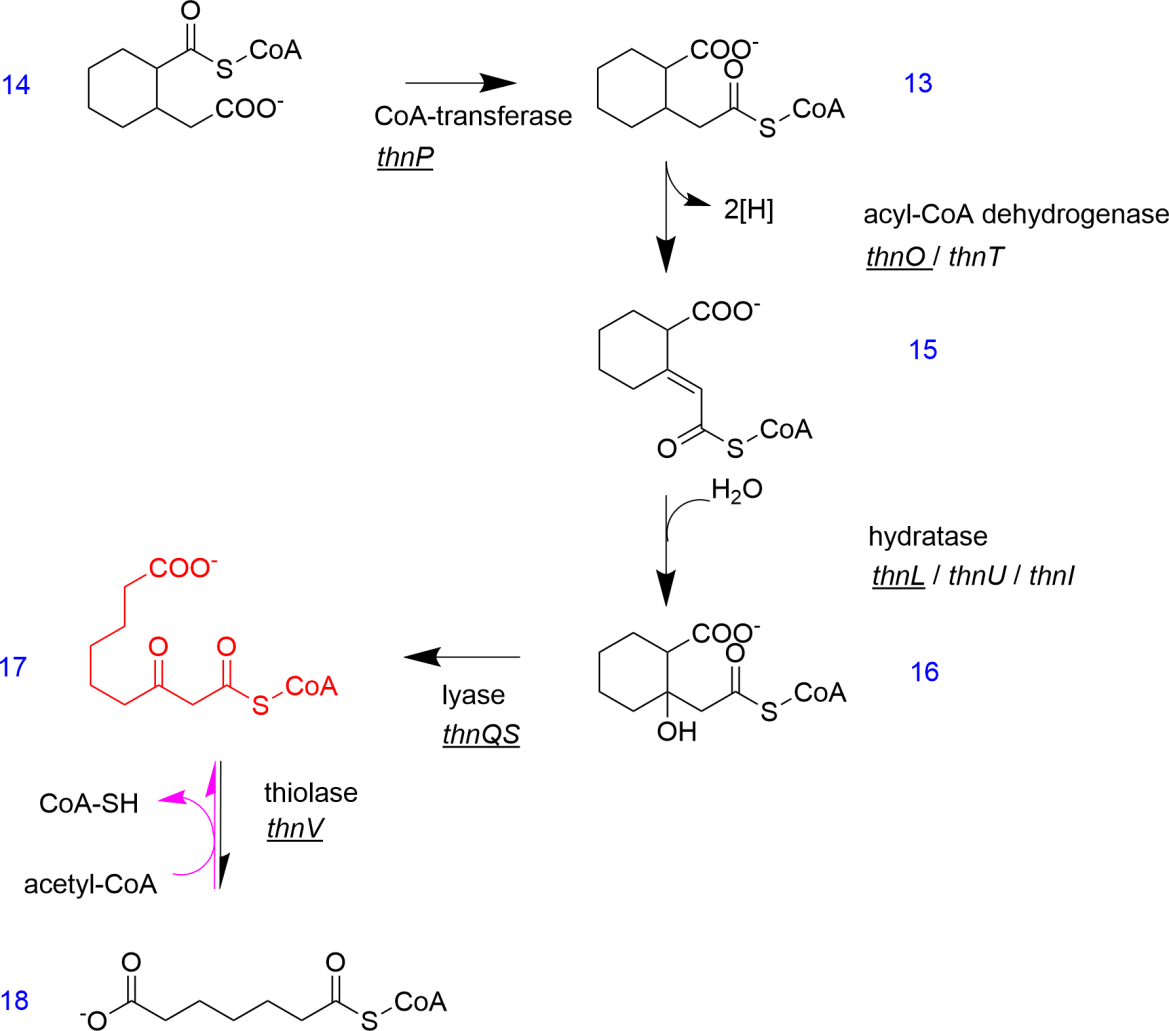
Cleavage reactions of the second naphthalene ring result in the formation of acetyl-CoA and pimeloyl-CoA. Metabolites and genes in black have been proven in enzyme assays, and the ones in red are hypothetical. Underlined genes represent enzymes specific to the reaction. Arrows in black indicate that the reactions have been measured in both N47 cell-free extracts and with heterologously expressed enzymes; reactions measured only with N47 cell-free extracts are indicated with a pink arrow. The conversion of 2-(carboxymethyl)cyclohexane-1-carboxyl-CoA (**14**) proceeds via 2-carboxycyclohexylacetyl-CoA (**13**), 2-carboxycyclohexylideneacetyl-CoA (**15**), 2-(1-hydroxy-2-carboxycyclohexyl)acetyl-CoA (**16**), 3-oxoazeloyl-CoA (**17**), and pimeloyl-CoA (**18**).

The expected next step is a thiolytic cleavage releasing acetyl-CoA as indicated by the metabolites 2-carboxycyclohexylacetyl-CoA or 2-(carboxymethyl)cyclohexane-1-carboxyl-CoA (compound **13** or **14** in Fig. 6), which has been measured in enzyme assays using crude cell extracts of N47 (101). This reaction is likely catalyzed by the gene product of *thnJ*, because *thnV*, another gene annotated as *β*-oxoacyl-CoA thiolase in the *thn*-operon, has been demonstrated to be responsible for the conversion of the metabolite 3-oxoazeloyl-CoA (compound **17** in Fig. 7) to pimeloyl-CoA (compound **18** in Fig. 7) in the downstream pathway.

### Cleavage of the second ring of naphthalene

The second ring-cleave in the further degradation pathway has been proposed based on the conversion of the CoA thioester of 2-(carboxymethyl)cyclohexane-1-carboxylic acid (compounds **13** and **14** in Fig. 7) in cell-free extracts of strains N47 and NaphS2 (107). The CoA-thioester can be located either at the acetyl residue or at the carboxyl residue, which is still unknown. However, the downstream conversion of 2-(carboxymethyl)cyclohexane-1-carboxyl-CoA (compound **14** in Fig. 7) starts with a CoA-transferase reaction since a racemic mixture of the CoA-thioester is converted into only one compound by N47 cell-free extracts (107). A discontinuous *in vitro* assay of the CoA-transferase was performed with heterologously expressed and purified enzymes from N47, demonstrating that the *thnP* gene product catalyzes the required intramolecular CoA-transfer (102). This allows for complete conversion of the CoA thioester of 2-(carboxymethyl)cyclohexane-1-carboxylic acid since only the isomer with the CoA thioester located at the acetyl side chain acts as the substrate of the following dehydrogenase. The confirmed function of the CoA-transferase ThnP demonstrates that the product of the first ring cleavage of naphthalene is 3-(2-[carboxymethyl]cyclohexyl)-3-oxopropionyl-CoA (compound **12** in Fig. 6), rather than 4-(2-carboxycyclohexyl)-3-oxobutyryl-CoA (compound **11** in Fig. 6). This subsequently leads to the formation of 2-(carboxymethyl)cyclohexane-1-carboxyl-CoA (compound **14** in Fig. 6) and the following coenzyme A transfer reaction.

The acyl-CoA dehydrogenases (ACADs) involved in anaerobic naphthalene degradation in strain N47 belong to the ACAD family and are encoded by the genes *thnO* and *thnT* in the *thn* operon (102). The two enzymes share 58% amino acid sequence similarity, and both ThnO and ThnT can catalyze the dehydrogenase reaction of 2-carboxycyclohexylacetyl-CoA (compound **13** in Fig. 7) introducing a double bond leading to 2-carboxycyclohexylideneacetyl-CoA (compound **15** in Fig. 7) (102). However, since ThnO has a 41-fold higher specific activity, it is most likely the responsible enzyme. ACAD-like enzymes are also involved in other anaerobic degradation pathways, for example, cyclohexanecarboxyl-CoA dehydrogenase and cyclohex-1-ene-1-carboxyl-CoA dehydrogenases participating in the fermentation of benzoate and crotonate by *Syntrophus aciditrophicus* (108).

The formation of a double bond in α,β-position of 2-carboxycyclohexylideneacetyl-CoA (compound **15** in Fig. 7) is followed by water addition in β-position producing a tertiary hydroxyl group. When the potential hydratase genes *thnA*, *thnM*, *thnL*, *thnU*, *thnH*, and *thnI* in the *thn*-operon were expressed heterologously, gene products ThnL, ThnU, and ThnI were all capable of converting the substrate. However, ThnL exhibited the highest specificity as a hydratase for this reaction, yielding 2-(1-hydroxy-2-carboxycyclohexyl)acetyl-CoA (compound **16** in Fig. 7) (Y. Kong and R. U. Meckenstock, unpublished results). This finding broadens the substrate spectrum of ThnL, indicating its role as a bifunctional hydrolase/enoyl-CoA hydratase, contrasting with previous studies that ThnL was exclusively a hydrolase targeting cyclic compounds (75). The product 2-(1-hydroxy-2-carboxycyclohexyl)acetyl-CoA now has a tertiary hydroxyl group which is not amenable to further beta-oxidation-like reactions because the hydroxyl group cannot be oxidized to a keto group.

Hence, we propose that the next step in the pathway is a lyase reaction cleaving the aliphatic bond between the C1 and C2 of the cyclohexane ring of 2-(1-hydroxy-2-carboxycyclohexyl)acetyl-CoA (compound **16** in Fig. 7). Indeed, the potential lyase gene products of *thnQ* and *thnS* in the *thn* operon were proven to be responsible for the lysis of 2-(1-hydroxy-2-carboxycyclohexyl)acetyl-CoA producing 3-oxoazeloyl-CoA (compound **17** in Fig. 7). Afterward, 3-oxoazeloyl-CoA was cleaved to pimeloyl-CoA (compound **18** in Fig. 7) and acetyl-CoA by a heterologously expressed thiolase encoded by *thn*V in the *thn-*operon (Y. Kong and R. U. Meckenstock, unpublished results). ThnV was identified using a combination of heterologously expressed lyase (ThnQS) and thiolase (ThnV), with the conversion of 2-(1-hydroxy-2-carboxycyclohexyl)acetyl-CoA (compound **16** in Fig. 7) to pimeloyl-CoA (compound **18** in Fig. 7) being monitored. The thiolysis of 3-oxoazeloyl-CoA is reversible, which was proven by assays with pimeloyl-CoA and acetyl-CoA in N47 cell-free extracts (107). The conversion of 2-(1-hydroxy-2-carboxycyclohexyl)acetyl-CoA to pimeloyl-CoA was measured with N47 cell-free extracts as well (Y. Kong and R. U. Meckenstock, unpublished results). The lyase reaction acting on 2-(1-hydroxy-2-carboxycyclohexyl)acetyl-CoA in N47 is comparable to the 3-hydroxy-3-methylglutaryl-CoA lyase LiuE involved in the leucine/isovalerate utilization pathway in *Pseudomonas aeruginosa*, which catalyzes the cleavage of 3-hydroxy-3-methylglutaryl-CoA to acetyl-CoA and acetoacetate (109).

We propose that the further steps of pimeloyl-CoA degradation will follow regular, known beta-oxidation reactions yielding three acetyl-CoA units that can be fully oxidized in the citric acid cycle or the Wood-Ljungdahl pathway (110).

## DEGRADATION OF PHENANTHRENE AND BIGGER PAHs

### Activation and CoA-ligation

In sulfate-reducing, phenanthrene-degrading enrichment cultures, phenanthrene is activated by carboxylation as shown by stable isotope labeling experiments with ^13^C-labeled bicarbonate buffer, analogous to naphthalene activation (22, 43, 44). In the enrichment culture TRIP_1, two gene clusters were found that encode a putative carboxylase with multiple subunits, similar to naphthalene carboxylases of strains N47 and NaphS2, and were detected during transcriptomic and proteomic analysis (39).

Carboxylation is immediately followed by ligation of phenanthroate to phenanthroyl-CoA (44). The substrate specificity of the enzyme 2-phenanthroate:CoA ligase, catalyzing the CoA-ligation reaction, allowed to deduce the natural product of the carboxylase reaction. All possible isomers of phenanthroic acid were purchased and used as substrates in enzyme assays of 2-phenanthroate:CoA ligase with cell-free extracts of the sulfate-reducing culture TRIP_1 and with purified phenanthroate:CoA ligase that was heterologously expressed in *E. coli*. In contrast to the 2-naphthoate:CoA ligase, the 2-phenanthroate:CoA ligase is highly specific; it can only act on 2-phenanthroate and on 3-phenanthroate with a much lower conversion rate, indicating that 2-phenanthroic acid is the product of phenanthrene carboxylation (74, 79).

Furthermore, CoA transferases could be involved in the conversion of 2-phenanthroic acid to 2-phenanthroyl-CoA, since a gene cluster encoding for such putative enzymes was identified in the neighborhood of the gene cluster coding for 2-phenanthroate:CoA ligase. CoA transferases are preferred by some microorganisms under energy-limited conditions, but there is no biochemical evidence for such reactions in anaerobic phenanthrene degradation so far (28).

### Reduction of the phenanthrene aromatic ring system

Metabolites were identified from culture supernatants and indicated a stepwise reduction of the aromatic ring system of phenanthroate to overcome the resonance energy, following the same principle as for naphthalene degradation. The metabolites were always two mass units heavier than the substrates, indicating a ring reduction of 2-phenanthroyl-CoA in two-electron steps, potentially starting from ring III of 2-phenanthroic acid, opposite to the carboxyl group (44). Hence, there is a high possibility that phenanthrene aromatic rings are dearomatized using enzymes similar to the ones involved in anaerobic naphthalene degradation.

In a proteogenomic analysis of culture TRIP_1, four oxidoreductase genes (PITCH_v1_a10001, PITCH_v1_aa1860005, PITCH_v1_a420108, and PITCH_v1_a190075) were identified belonging to the old-yellow enzyme family similar to the type III aryl-CoA reductases, 2-naphthoyl-CoA reductase (NCoA-R) and 5,6-dihydro-2-naphthoyl-CoA reductase (DHNCoA-R) (39). Recently, we expressed the four oxidoreductase genes heterologously in *E. coli* to validate their function and to identify which enzyme is responsible for each reduction step of the phenanthrene aromatic ring system (N. A. Samak and R. U. Meckenstock, unpublished results). The purified gene product of PITCH_v1_a10001 catalyzed the reduction of 2-phenanthroyl-CoA to dihydro-2-phenanthroyl-CoA. PITCH_v1_a1860005 catalyzed the reduction of the dihydro-2-phenanthroyl-CoA to tetrahydro-2-phenanthroyl-CoA and directly converted the tetrahydro-2-phenanthroyl-CoA to hexahydro-2-phenanthroyl-CoA. After the formation of the hexahydro-2-phenanthroyl-CoA, PITCH_v1_a420108 catalyzed the further reduction to octahydro-2-phenanthroyl-CoA. The fourth oxidoreductase enzyme, PITCH_v1_a190075, catalyzed the last reduction step from octahydro-2-phenanthroyl-CoA to decahydro-2-phenanthroyl-CoA. Notably, the complete reduction of the three aromatic rings of 2-phenanthroyl-CoA was accomplished in the absence of ATP, prompting questions on the thermodynamic feasibility of this process. The four oxidoreductases responsible for the reduction of 2-phenanthroyl-CoA were also characterized and contained stoichiometric amounts of FMN, FAD, and iron-sulfur clusters, similar to the type III aryl-CoA reductases involved in anaerobic naphthalene degradation. Hence, the reduction of phenanthroyl-CoA seems to follow the same principles as the reduction of 2-naphthoyl-CoA, with the main difference that no ATP-dependent reduction step is involved. Further steps of anaerobic phenanthrene degradation, including the ring cleavage reactions, are still unclear.

Anaerobic phenanthrene degradation was also studied in strain PheN1, which was isolated from a 5:1:1 ratio mixture of petroleum-contaminated soil, coking sludge, and domestic sludge, respectively (46). Strain PheN1 was published as a phenanthrene-degrading denitrifier growing with phenanthrene as a sole carbon source. It is phylogenetically related to *Achromobacter denitrificans* and reduces nitrate to nitrite during the anaerobic degradation of phenanthrene. Interestingly, activation of phenanthrene in this strain is claimed to happen both via carboxylation and methylation, producing 2-phenanthroic acid and 2-methylphenanthrene, respectively, which is rather unlikely because the cells should only use one of the two options. However, a similar problem arose during the early studies of anaerobic naphthalene degradation where a metabolite of 2-methylnaphthalene degradation appeared (naphthyl-2-methyl-succinyl-CoA) (52) suggesting a methylation of naphthalene to 2-methylnaphthalene. It was found out later that the substrate naphthalene contained traces of 2-methylnaphthalene which were activated by fumarate addition and produced the misleading metabolite. Hence, ambiguous results in metabolite analyses might stem from impurities of the used substrates (52). Moreover, in strain PheN1, the downstream degradation revealed the conversion to benzene compounds and cyclohexane derivatives. Downstream degradation metabolites detected during the degradation were 2-hydroxy-5-methylacetophenone, p-cresol, 4-methyl-cyclohexanol, and succinic acid (46). Hence, further biochemical studies are needed to understand nitrate-dependent PAH degradation.

Under anoxic conditions, the methane-oxidizing denitrifying bacterium *Candidatus* Methylomirabilis oxyfera, alongside an alkane-oxidizing γ-proteobacterium (HdN1), may generate oxygen by breaking down nitric oxide (NO) through a disproportionation process (111, 112). Genes potentially responsible for this NO disproportionation into oxygen are commonly found in environments contaminated with PAHs as well as in systems treating wastewater to remove nitrogen (113). Hence, the degradation of PAHs with nitrate as electron acceptor might also proceed through aerobic reactions due to the production of very low amounts of oxygen through NO disproportionation.

Metabolite analyses of the nitrate-reducing *Pseudomonas* strain JP1 degrading bigger PAHs did not show the same metabolite patterns as the above-mentioned studies with sulfate-reducers (114). The degradation metabolites observed during the anaerobic degradation of benzo[a]pyrene by strain JP1 surprisingly indicated a direct ring cleavage without prior carboxylation, producing the metabolites 1,12-dimethyl benz[a]anthracene, 7,8,9,10-tetrahydrobenz[a]pyrene, 5-ethylchrysene, chrysene/benz[a]anthracene, three-ring PAHs, and two-ring PAHs, according to GC/MS analysis. Moreover, metabolite analysis of benz[a]anthracene degradation by the bacterium *Pseudomonas* JP1 revealed 2,3-dimethylphenanthrene and 2-methylphenanthrene or 2-methylanthracene/1-methylanthracene. The same bacterium anaerobically degraded phenanthrene and anthracene to produce the metabolites 1,2,3,4-tetrahydro-4-methyl-4-phenanthrenol and 1-anthraquinonecarboxylic acid, respectively (114). According to the principles of anaerobic degradation of PAHs outlined above for sulfate reducers, it is unlikely that metabolites without carboxyl or hydroxyl groups are generated.

## THERMODYNAMIC CONSIDERATIONS

Thermodynamic calculations show that the activation of naphthalene to 2-naphthoic acid is slightly endergonic under standard conditions with Δ*G*^0′^ = +14.2 kJ/mol. The overall energy yield of the complete oxidation of naphthalene to CO_2_ and assuming NADH as electron acceptor is −136.8 kJ/mol under standard conditions (20, 115), which allows for building only 2–3 ATP. The estimated ATP yield per mole of naphthalene consumed with sulfate as an electron acceptor is 4.1 ATP per mole (28).

In N47, there are nearly 20 enzyme reactions necessary to degrade naphthalene to acetyl-CoA, which is then fed into the central metabolism. Considering the small energy conservation, nearly all of the enzymatic reactions have to run at thermodynamic equilibrium, making them very slow. Degradation of bigger PAHs such as phenanthrene leads to slightly higher energy conservation, e.g. 4.9 mol ATP per mole of phenanthrene for sulfate reducers (28), but the pathway also needs more reactions. The little energy conservation and the need to run most reactions close to the thermodynamic equilibrium might be the reason why anaerobic PAH degraders grow so slowly with doubling times of 8 days for the naphthalene-degrading strain N47 and around two weeks for the phenanthrene-degrading culture TRIP_1 (44). It can be expected that organisms degrading bigger PAHs with more than three rings might grow even slower, if microorganisms can grow under similar conditions, but the evidence for degradation of such bigger PAHs is scarce.
